# The impact of genetic modified Ma bamboo on soil microbiome

**DOI:** 10.3389/fmicb.2022.1025786

**Published:** 2022-11-01

**Authors:** Kai Wang, Mengxia Liu, Changyang Cai, Shifeng Cai, Xiangqing Ma, Chentao Lin, Qiang Zhu

**Affiliations:** ^1^College of Forestry, Fujian Agriculture and Forestry University, Fuzhou, China; ^2^Basic Forestry and Proteomics Center, College of Forestry, Fujian Agriculture and Forestry University, Fuzhou, China; ^3^YouXi National Forestry Station, YouXi, China

**Keywords:** bamboo, GMO, microbiota, microbial community function, rhizosphere

## Abstract

Evaluating the potential alteration of microbial communities is a vital step for biosafety of genetic modified plants. Recently, we have produced genetic modified Ma bamboo with increased cold and drought tolerance by anthocyanin accumulation. In this work, we aim to study the potential effects on microbial communities in rhizosphere soils during the cultivation of genetic modified bamboo. Rhizosphere and surrounding soil were collected at 3-month post-transplant. The amplicon (16S rDNA and ITS1) were sequenced for analysis of bacterial and fungal communities. Multiple software and database (Picrust2, FAPROTAX and FUNGulid) were applied to predict and compare the microbial functions involving basic metabolisms, nitrogen usage and presence of plant pathogens. There were no substantial change of the structure and abundance of rhizosphere soil microbial communities between genetic modified and wild type bamboo. For the surrounding soil, the bacterial biota α-diversity increased (chao1: 1,001 ± 80–1,276 ± 84, observed species: 787 ± 52–1,194 ± 137, PD whole tree: 75 ± 4–117 ± 18) and fungal biota α-diversity decreased (chao1: 187 ± 18–145 ± 10) in samples of genetic modified bamboo compared to those of wild type bamboo. The microbiota predicted functions did not change or had no negative alteration between genetic modified and wild type bamboo, in both rhizosphere and surrounding soils. As a conclusion, the growth of genetic modified bamboo had no substantial change on rhizosphere soil microbial communities, while minor alteration on bamboo surrounding soil microbial communities with no harmful effects. Moreover, the genetic modified bamboo had no negative effect on the predicted functions of microbiota in soil.

## Introduction

Since the first commercialization of genetic modified (GM) tomato in 1996, the plantation area of GM plants is increasing dramatically. The GM plants have been cultivated more than 190 million hectares till 2021, benefiting 1.95 billion people in main GM plant production countries (United States, Brazil, Argentina, Canada, and India; Verma et al., 2021). The usage of GM crops has been proposed as the potential approach to solve the potential crisis of food shortage, climate change, sustainable development, and environment protection. However, it has been a long debate for the biosafety issue of cultivation and application of GM plants and their products.

There are mainly four aspects of GM plant biosafety, including the steady expression of foreign genes, evasion of foreign genes, impact on soil microbial abundance and interruption of soil microbial diversity (Lu et al., 2018; Wang et al., 2018; Li et al., 2022). Plant rhizosphere and endospheric microbes play vital roles in soil ecology and plant health, through participating the element exchange between plants and soil, or producing plant beneficial molecules and/or anti-stress compounds (Mendes et al., 2011; Lareen et al., 2016; Finkel et al., 2020; Wang et al., 2021). Some studies have shown that GM plants reduce the soil microbial diversity (Guan et al., 2016; Quemada, 2022). On the other hand, more studies illustrated that cultivation of GM plants had no or minor effects on soil microbial communities (Faragová et al., 2011; Guan et al., 2016; Li et al., 2022; Quemada, 2022). Thus, there is no golden standard to judge the effect of GM plants on soil microbiota, and we need to evaluate the consequence in a plant-specific and gene-specific manner. The development of amplicon sequencing and metagenome sequencing enable us to monitor the alteration of microbes in a culture-free way, for example, the structure and abundance of total microbial communities can be assessed by high throughput sequencing methods (Tyson et al., 2004; Gonzalez et al., 2012).

Bamboo, which belongs to the grasses family (Poaceae), is an important forestry resource in the world due to their great economic, social, environmental and culture values (Ramakrishnan et al., 2020). Bamboo products were widely used in our daily life, and the global bamboos market size was valued at 53.28 billion USD in 2020, and is expected to expand at a compound annual growth rate (CAGR) of 5.7% from 2021 to 2028.^1^ Moreover, bamboo forestry has excellent carbon fixation ability (Wang et al., 2021), and the demands for economically important bamboo species are increasing. Whereas, most giant bamboo species including Ma bamboo (*Dendrocalamus latiflorus* Munro), which provides valuable natural construction materials and high quality young bamboo shoots as food and acts as one of the most important giant bamboo species in South Asia, are grown in the warm and moist tropical and subtropical area, and their distributions and development are largely affected by abiotic stresses, mainly cold and drought (Liu et al., 2020; Wang et al., 2022). The question on how to improve their abiotic stress tolerance remains challenging for bamboo basic research and plantation. Due to its long flowering periods, it’s quite difficult to make agronomic improvement through classical breeding. Genetic engineering is an attractive approach for bamboo researchers, whereas only two reports showed the generation of cold-tolerant bamboos hitherto (Qiao et al., 2014; Xiang et al., 2021). Recently, we successfully established the Ma bamboo genetic transformation protocol by using young shoot as the explant (Ye et al., 2017), and produced purple Ma bamboo through ectopically expressing a maize *Lc* (leaf color) gene (Xiang et al., 2021). The transgenic bamboo showed increased accumulation of anthocyanin, improved cold and drought tolerance. The horticultural values and agronomic traits are substantially improved, indicating that the GM bamboo could be potentially applied in practice. Although the gene escaping in GM Ma bamboo *via* pollen has low risk because of its long flowering period (normally 120 years), the impact of GM Ma bamboo on soil ecosystem is a major concern over risk associated with their commercial release. Therefore, the biosafety issue of its cultivation remains to be investigated.

In this study, we aim to evaluate the ecological risk of GM Ma bamboo expressing maize *Lc* gene on the microbial communities of both rhizosphere and surrounding soil in the restricted greenhouse condition. By amplifying 16S rDNA and ITS1 amplicons, we compared the abundance, diversity and function of both bacterial and fungal communities. Our results indicated that there were no substantial differences in the microbial abundance and diversity of rhizosphere soil between GM and wild type bamboos. Whereas, GM bamboo had increased bacterial abundance and diversity, as well as decreased fungal abundance and diversity in the surrounding soil. Moreover, there was no difference between GM and wild type bamboo samples related to basic metabolism, genetic processing, environmental processing and pathogenicity to plants based on the predicted functions. In summary, we concluded that the cultivation of GM Ma bamboo overexpressing maize *Lc* gene has no negative impact on soil microbial communities.

## Materials and methods

### Cultivation of bamboo and soil collection

GM bamboo with overexpressed maize *Lc* gene was generated as previously described (Xiang et al., 2021). Based on the policy in China, GM and wild type bamboo were grown in the restricted greenhouse condition (temperature: 22°C, light/dark: 16/8 h, light intensity: 100 μmol m^−2^ s^−1^, humidity: 70%). Soil used in this study was the mixture of 70% of Danish peat soil (0–6 mm, pH 6) and 30% vermiculite. Tissue-culture seedlings at the same age were transferred into pots filled with mixture soil. The pots were randomly placed in greenhouse. The mixture soil we used is beneficial for the rooting and growth of tissue culture seedlings, as well as to maintain water content. Sampling sites of bamboo surrounding soil were from the double diagonal of pots, 5–10 cm away from bamboo rhizosphere. Bamboo was planted in pots (10 × 10 × 10 cm) with soil for 3 months before collecting soil samples. Surrounding soil was harvested by collecting the soil in pot corners that 5–10 cm away from root. The samples from corners were merged into one sample. Rhizosphere soil was collected by gently brushing the soil that closely attached to bamboo roots, after separating the root system and soil. Three biological repeats were conducted for each treatment. Totally we conducted four treatments: rhizosphere soil of GM bamboo, rhizosphere soil of WT bamboo, surrounding soil of GM bamboo, and surrounding soil of WT bamboo. Soil samples in sterile 50 ml Eppendorf tube were immediately frozen in liquid nitrogen, and were kept in −80°C until further process.

### Amplicon sequencing

Soil microbial total DNA was isolated by following the procedure of TIANamp Soil DNA Kit (TIANGEN, China). The integrity of isolated DNA was examined by 1% agarose gel electrophoresis. Total DNA concentration was diluted into 10 ng/μl with ddH_2_O. Total amount of 30 ng DNA was used as template for PCR, with 1 μl forward and reverse primers (16S rDNA V3V4, forward, ACTCCTACGGGAGGCAGCAG, reverse: GGACTACHVGGGTWTCTAAT; ITS1, forward: CTTGGTCATTTAGAGGAAGTAA, reverse: TGCGTTCTTCATCGATGC) (5 μM), 3 μl BSA (2 ng/μl), 12.5 μl 2 x Taq Plus Master Mix, and adjusted ddH_2_O as 25 μl PCR system. 16S rDNA V3V4 region was amplified for 30 cycles (94°C for 30s, 50°C for 30s, 72°C for 60s). ITS1 region was amplified for 34 cycles (94°C for 30s, 55°C for 30s, 72°C for 60s). The size of PCR products was checked with 1% agarose gel electrophoresis, after which PCR products were purified with Agencourt AMPure XP kit (Beckman Coulte, United States). Purified PCR products were then sequenced with Miseq platform.

### Sequence data analysis

A brief workflow of data analysis was provided in Supplementary Figure 1. Raw reads were merged (minimum overlap 10 bp, error 0.1) with Pear v0.9.6 (Zhang et al., 2013) and Flash v1.20 (Magoč and Salzberg, 2011) into raw tags. Raw tags were filtered with Trimmomatic v0.36 and Pear v0.9.6 (*Q* < 20 in 50-base sliding windows, minimum length setting 120 bp) to remove low quality reads (Zhang et al., 2013; Bolger et al., 2014). Clean tags were grouped into OTUs (Operation Taxonomic Units) using UPARSE with 97% similarity (Edgar, 2013). Representative sequence of each OTU was assigned into multiple taxonomic levels with Mothur (Schloss et al., 2009) by blast against 16S Silva database (release 138) (Quast et al., 2012) and ITS Unite database (release 8.2). Chimera tags and Singleton tags were removed with Uchime algorithm based on Gold database (Edgar et al., 2011) to get effective tags. Normalization was carried out to effective tags of each sample using the lowest count number. Subsequent analysis was conducted based on normalized effective tags. The specific and shared OTUs among samples were visualized with Venn diagram in R.

Rarefaction curves of bacterial and fungal OTUs were produced with vegan package in R based on OTUs clustering. The chao1, observed species, PD whole tree and Shannon index of mean values from three biological repeats were analyzed to represent microbial community α–diversity with Qiime v1.8.0 (Caporaso et al., 2010). *T*-test of above indexes were performed in R to evaluate the differences between parallel samples. PCA (Principal Component Analysis) of bacterial and fungal OTUs were conducted for comparison of microbial community structures between GM and wild type samples.

The following analysis were conducted with mean values from three biological repeats. Picrust2 (Phylogenetic Investigation of Communities by Reconstruction of Unobserved States 2) was applied to predict the potential function of bacterial communities, with the information of KEGG pathway database (Douglas et al., 2020). To further explore the effect of GM bamboo on ecological functions, functional group assignments of bacterial relative OTU taxonomic table were performed with command collapse_table.py of FAPROTAX 1.2.5 (Louca et al., 2016). Visualization of relative abundance of selective functional groups was performed with package ggplot2 in R. Significant difference between GM and wild type bamboo was conducted with *t*-test in R. FUNGulid database (Nguyen et al., 2016) was used to annotate fungal OTU functions in species level, especially for plant pathogens. Since pathogenicity in fungi genus level are not reliable, we selected group plant pathogen only in species level.

## Results

### Soil microbiota amplicon sequencing information

**Figure 1:**
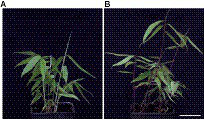
The 3-month old wild type Ma bamboo **(A)** and genetic modified Ma bamboo with overexpression of maize *Lc* (leaf color) gene **(B)** cultivated in soil pot at greenhouse condition. Photos were taken right before soil sample collection.

GM bamboo with overexpression of maize *Lc* gene exhibited purple color (Figures 1A,B; Xiang et al., 2021). The genomic DNA of soil microbes were isolated with high quality from samples of root surrounding soil and rhizosphere soil. The amplicons of microbiota from the wild type bamboo rhizosphere soil (WT-R), wild type bamboo surrounding soil (WT-S), GM bamboo rhizosphere soil (GM-R), and GM bamboo surrounding soil (GM-S) were sequenced. By sequencing the bacterial 16S rDNA V3-V4 region and fungal ITS1 region, we obtained 1,273,098 raw tags and 1,245,636 clean tags for 16S rDNA, as well as 1,534,084 raw tags and 1,272,287 clean tags for ITS1 for 12 samples in total. The distribution of clean tags of 16S rDNA V3-V4 region was 99.95% in 400–440 bp. The length of ITS1 clean tags was in 0–540 bp, with 83.18% in 200–360 bp. The sequence counts of all samples for 16S rDNA ranged from 55,509 to 138,683, with average of 97,892 ± 24,049 (mean ± standard deviation), and 43,922–149,710 (105,569 ± 34,879) for ITS1 sequence counts. The smallest sample sizes (55,509 for 16S rDNA and 43,922 for ITS1) were applied for random normalization for further analysis. Goods coverage were 100% for all samples. The OTUs had no obvious growth as the number of reads increased (Supplementary Figure 2), indicating the qualities of the sampled reads were satisfactory for the analysis of species richness. Representative amplicon sequence of each OTU was listed in Supplementary Files 1, 2.

### Soil microbial diversity and structure

**Figure 2:**
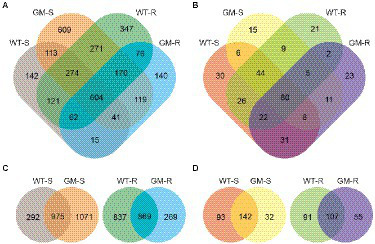
Venn diagrams of OTU grouping of bacterial biota **(A)** and fungal biota **(B)**, with combined rhizosphere and surrounding soil samples. Venn diagrams of OTU grouping of bacterial biota, with separated comparison of rhizosphere and surrounding soil samples **(C)**, as well as those for fungal biota **(D)**. WT-S: Wild Type Surrounding soil, GM-S: Genetic Modified Surrounding soil, WT-R: Wild Type Rhizosphere soil, GM-R: Genetic Modified Rhizosphere soil.

In total, 3,104 bacterial OTUs and 333 fungal OTUs were identified in this study. The 60.1% of bacterial OTUs and 73.3% of fungal OTUs were shared in at least two groups. The number of OTUs that uniquely found in one group were variable for both bacteria and fungi (Figures 2A,B). The unique bacterial OTUs of GM-S samples had relative high proportion (1,071 OTUs, 45.8%), compared to those of WT-S (292 OTUs, 12.5%). For the rhizosphere soil, however, the GM bamboo (269 OTUs, 13.6%) has less unique bacterial OTUs than wild type bamboo (837 OTUs, 42.4%; Figure 2C). As to the fungal biota, both the surrounding and rhizosphere soil of GM bamboo have less unique OTUs than those of wild type bamboo (Figure 2D). The assignment of three most abundant bacterial OTUs for WT-S were *Bacillus* sp. CMAA1185, uncultured Coxiellaceae bacterium and *Acinetobacter guillouiae* CIP 63.46; for WT-R were uncultured Coxiellaceae bacterium, uncultured Verrucomicrobia bacterium and *Devosia* sp.; for GM-S were uncultured Acidobacteria bacterium, *Acidibacter* sp., and *Devosia* sp.; for GM-R were uncultured Coxiellaceae bacterium, *Acinetobacter guillouiae* CIP 63.46 and uncultured Acidobacteria bacterium. Uncultured Coxiellaceae bacterium and *Devosia* sp. were the common abundant bacterial among four groups. Similarly, the assignment of most three abundant fungal OTUs for WT-S were Chytridiomycota fungus, Rozellomycotina cls Incertae sedis GS05 fungus and unidentified fungus; for WT-R were *Rhodotorula mucilaginosa*, unidentified fungus and *Wallemia tropicalis*; for GM-S were *Cladosporium halotolerans*, *Trichoderma* sp. and Rozellomycotina cls Incertae sedis GS05 fungus; for GM-R were *Cladosporium halotolerans*, Sordariales fungus and unidentified fungus.

**Figure 3:**
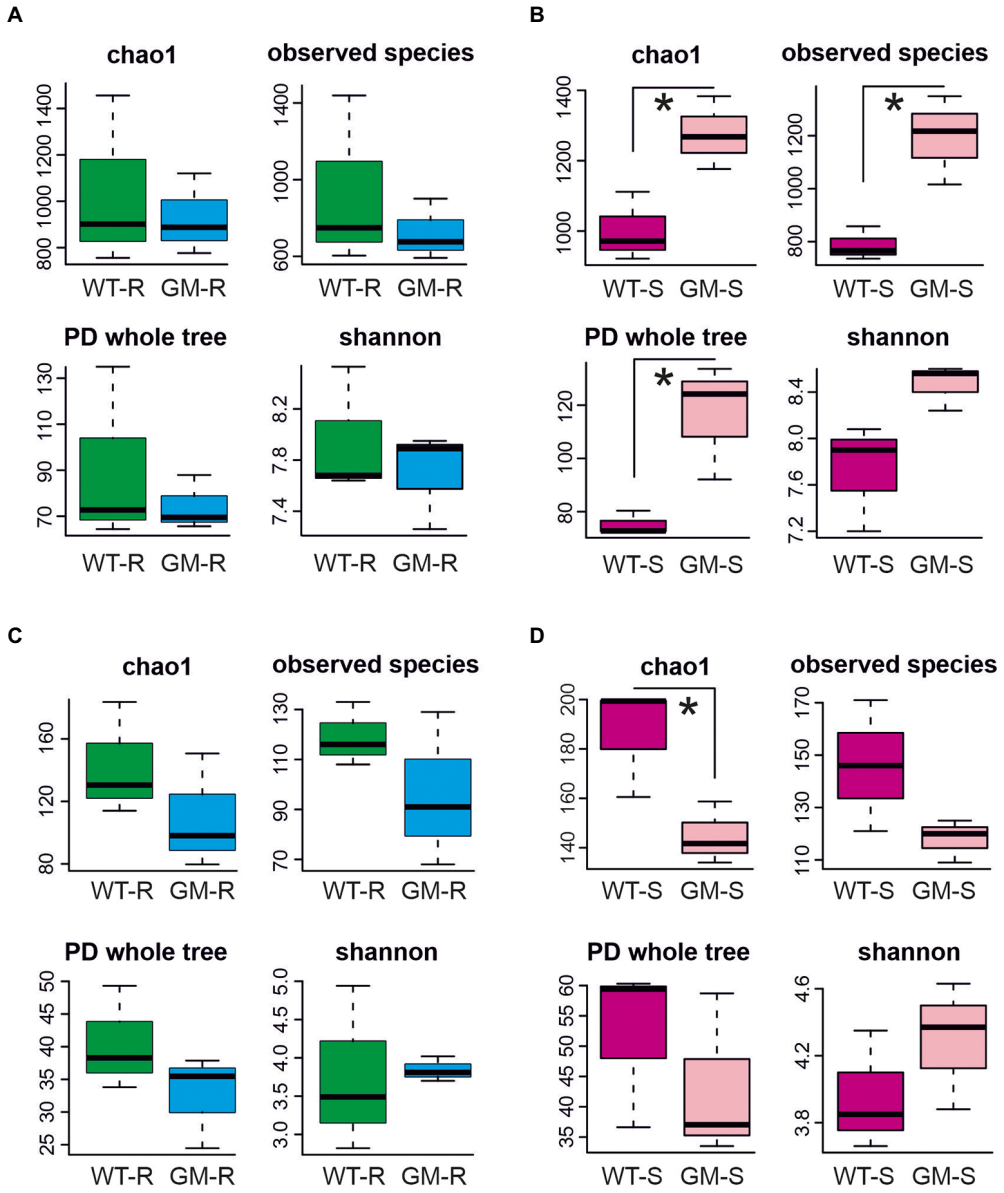
The indexes (chao1, observed species, PD whole tree, shannon) of α-diversity of bacterial communities in rhizosphere soil **(A)**, of bacterial communities in surrounding soil **(B)**, of fungal communities in rhizosphere soil **(C)**, of fungal communities in surrounding soil **(D)**. WT-R, Wild Type Rhizosphere soil; GM-R, Genetic Modified Rhizosphere soil; WT-S, Wild Type Surrounding soil; GM-S, Genetic Modified Surrounding soil. *p < 0.05.

**Figure 4:**
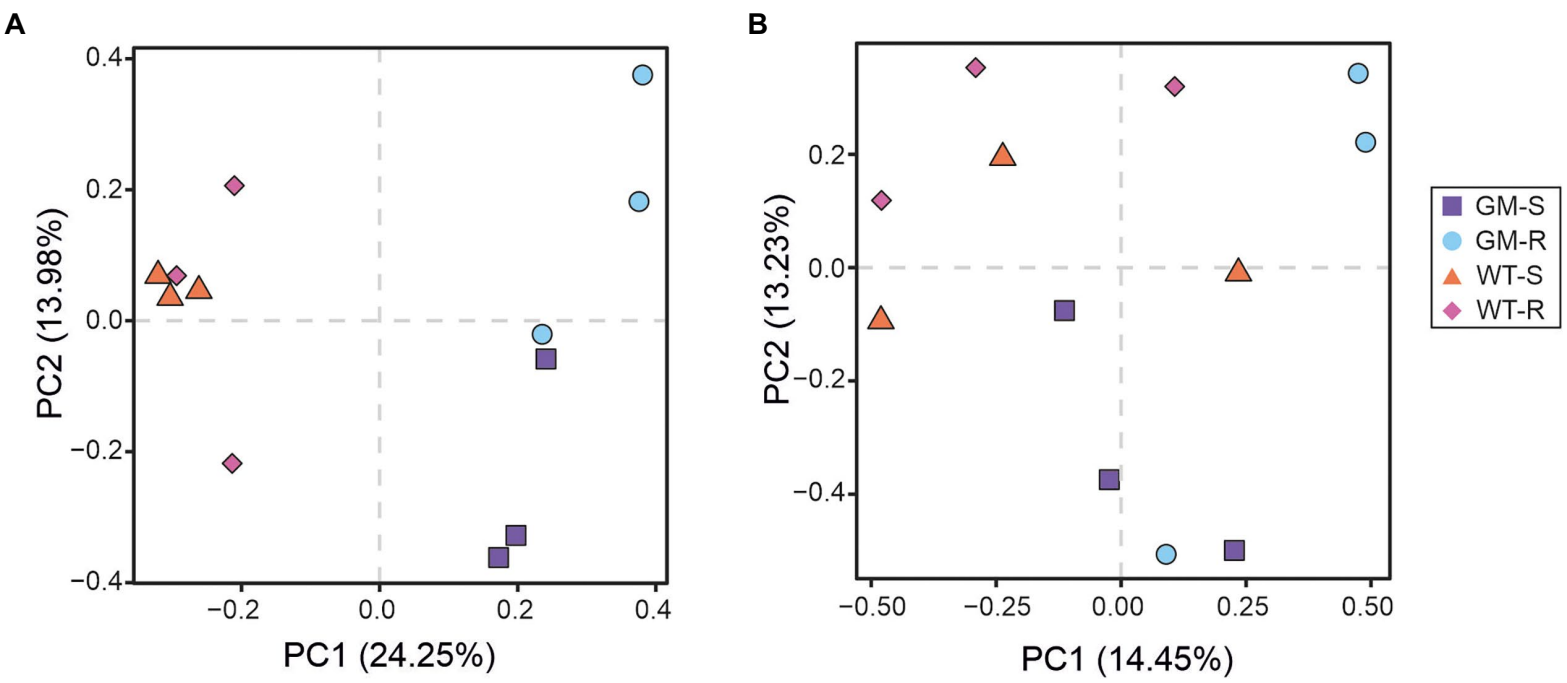
Principal component analysis of the bacterial community OTUs **(A)** and fungal community OTUs **(B)**. GM-S, Genetic Modified Surrounding soil; GM-R, Genetic Modified Rhizosphere soil; WT-S, Wild Type Surrounding soil; WT-R, Wild Type Rhizosphere soil.

Microbial OTUs were further assigned into 329 bacterial species and 133 fungal species. According to the results from Shannon and Rarefaction analysis, all of the soil microbial diversity in samples were captured with our sequence depth. There was no significant difference in α-diversity of rhizosphere-soil bacteria biota between wild type and GM bamboo plants, based on the analysis of chao1, observed species, PD whole tree and Shannon indexes (Figure 3A). As to the surrounding soil, however, the soil microbiota of GM bamboo had a higher diversity than those of wild type bamboo, with significant differences in chao1 (*p* < 0.05), observed species (*p* < 0.05), PD whole tree (*p* < 0.05; Figure 3B). For the rhizosphere soil fungi biota, the α-diversity between wild type and GM bamboo had no significant difference (Figure 3C), which was the same with the rhizosphere soil bacteria biota. The surrounding-soil fungi biota of GM bamboo had lower α-diversity than those of wild type bamboo, as suggested by significant difference in chao1 but not from the other indexes (Figure 3D). PCA analysis suggested that GM bamboo associated bacteria biota formed a group, which was distinct from the group composed by wild type bamboo associated bacteria biota in the view of PC1 (24.25%; Figure 4A). Surprisingly, the groups of GM bamboo associated and wild type associated fungi biota were not separated in neither PC1 nor PC2 (Figure 4B).

**Figure 5:**
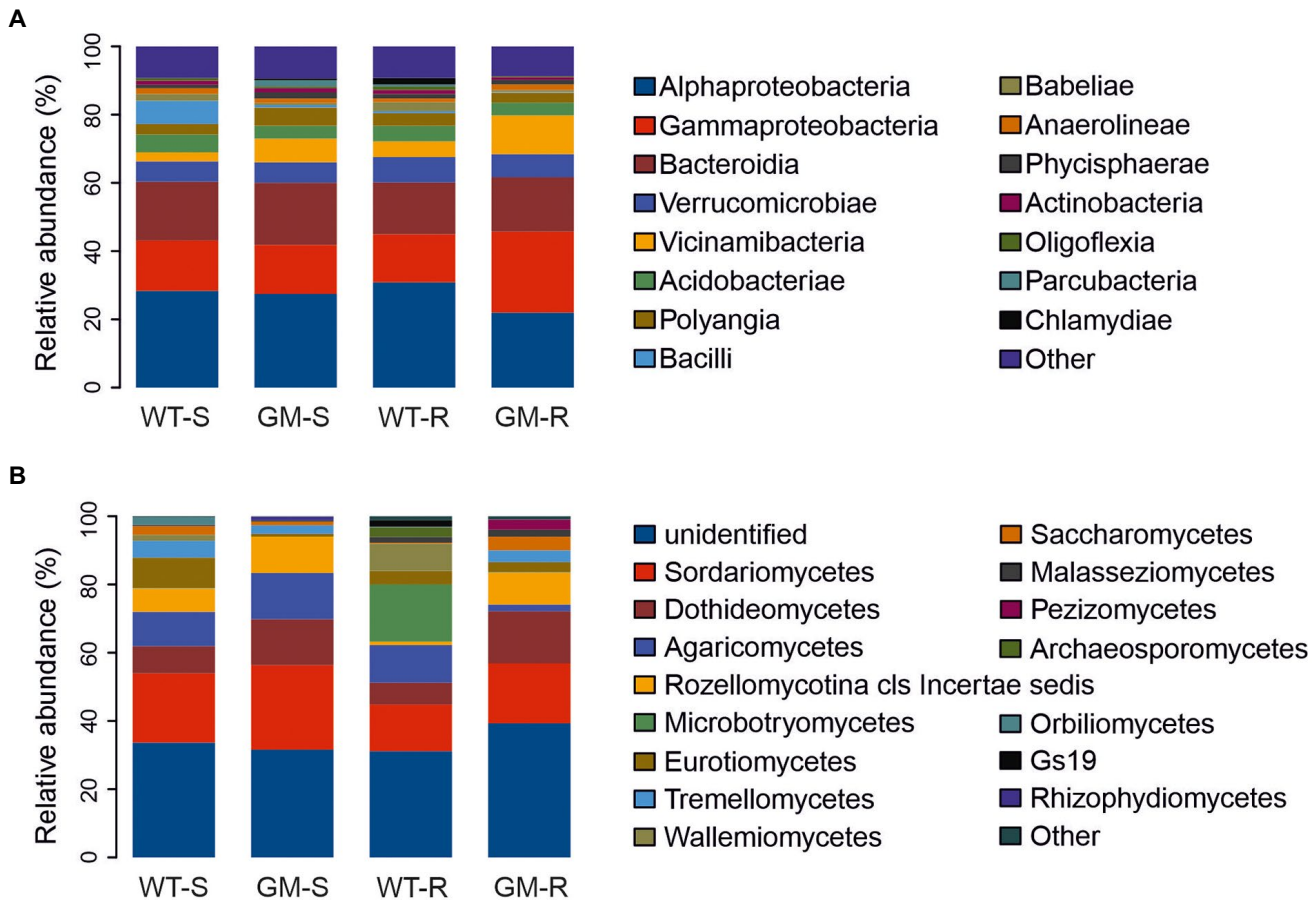
Relative abundance of bacterial communities **(A)** and fungal communities **(B)** in taxonomic class level. WT-S, Wild Type Surrounding soil; GM-S, Genetic Modified Surrounding soil; WT-R, Wild Type Rhizosphere soil; GM-R, Genetic Modified Rhizosphere soil.

The taxonomic abundance of soil bacteria and fungi were compared in multiple taxonomic levels. Interestingly, the overall bacterial taxonomic abundances were relatively conserved in four groups, as indicated in class level, except the enrichment of Gammaproteobacteria and Vicinamibacteria in GM bamboo rhizosphere group (Figure 5A). On the other hand, the fungal taxonomic abundances among groups were rather divergent, including the top abundant classes Sordariomycetes, Dothideomycetes, Agaricomycetes, etc. (Figure 5B).

### Functional prediction

**Figure 6:**
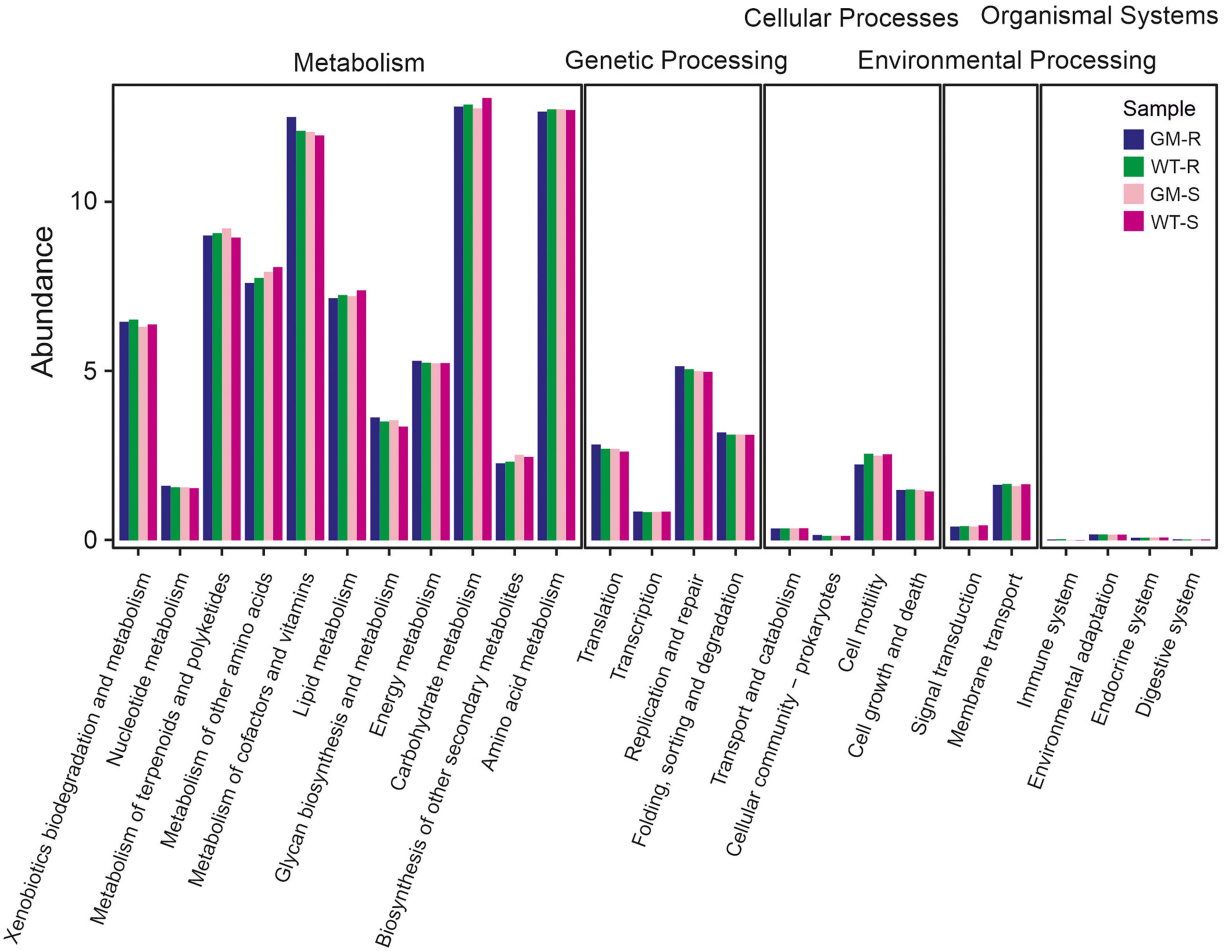
The abundance of predicted functions of bacterial communities involving metabolism, genetic processing, cellular processes, environmental processing, organismal systems among four different group of samples. Picrust2 was used to predict function of 16S rDNA markers. GM-R, Genetic Modified Rhizosphere soil; GM-S, Genetic Modified Surrounding soil; WT-R, Wild Type Rhizosphere soil; WT-S, Wild Type Surrounding soil.

**Figure 7:**
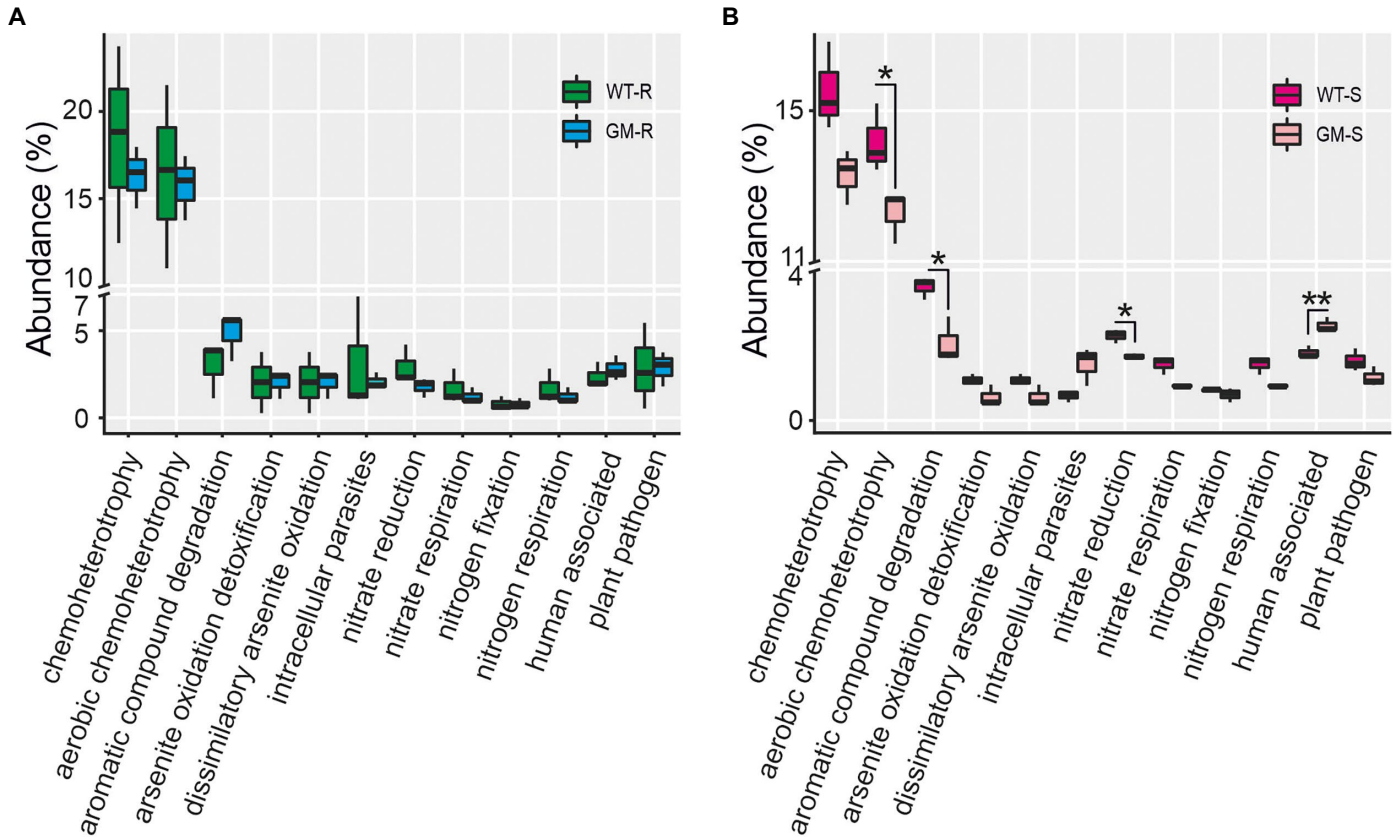
The abundance of predicted functions of bacterial communities using FAPROTAX for rhizosphere soil **(A)** and surrounding soil **(B)**. WT-R, Wild Type Rhizosphere soil; GM-R, Genetic Modified Rhizosphere soil; WT-S, Wild Type Surrounding soil; GM-S, Genetic Modified Surrounding soil.

To investigate the potential changes of soil microbiome, we compared the functional abundances of soil bacteria biota based on 16S rDNA and fungi biota on ITS1 markers. Picrust2, FAPROTAX and FUNGulid were applied for prediction of bacterial functions, offering an understanding of microbiota itself and the potential interaction with host bamboo. By closely examining the KEGG orthologs in microbial metabolism, genetic processing, cellular processes, environmental processing and organismal systems, we found that the functional abundances of all four groups kept at similar levels. Metabolism and genetic processing accounted for the majority of function abundance, with comparable values among all of groups (Figure 6). FAPROTAX functional prediction with 16S rDNA revealed that 710 out of 3,104 bacterial OTUs (22.8737%) were assigned to at least one functional group, and 36 functional groups were assigned (with ≥5 OTUs). Chemoheterotrophy and aerobic chemoheterotrophy were the most abundant functions (Figures 7A,B). For bamboo rhizosphere soil, there was no significant difference in functional abundance related to chemoheterotrophy, aerobic chemoheterotrophy, aromatic compound degradation, arsenite oxidation detoxification, dissimilatory arsenite oxidation, intracellular parasites, nitrate reduction, nitrate respiration, nitrogen fixation, and nitrogen respiration (Figure 7A).

**Figure 8:**
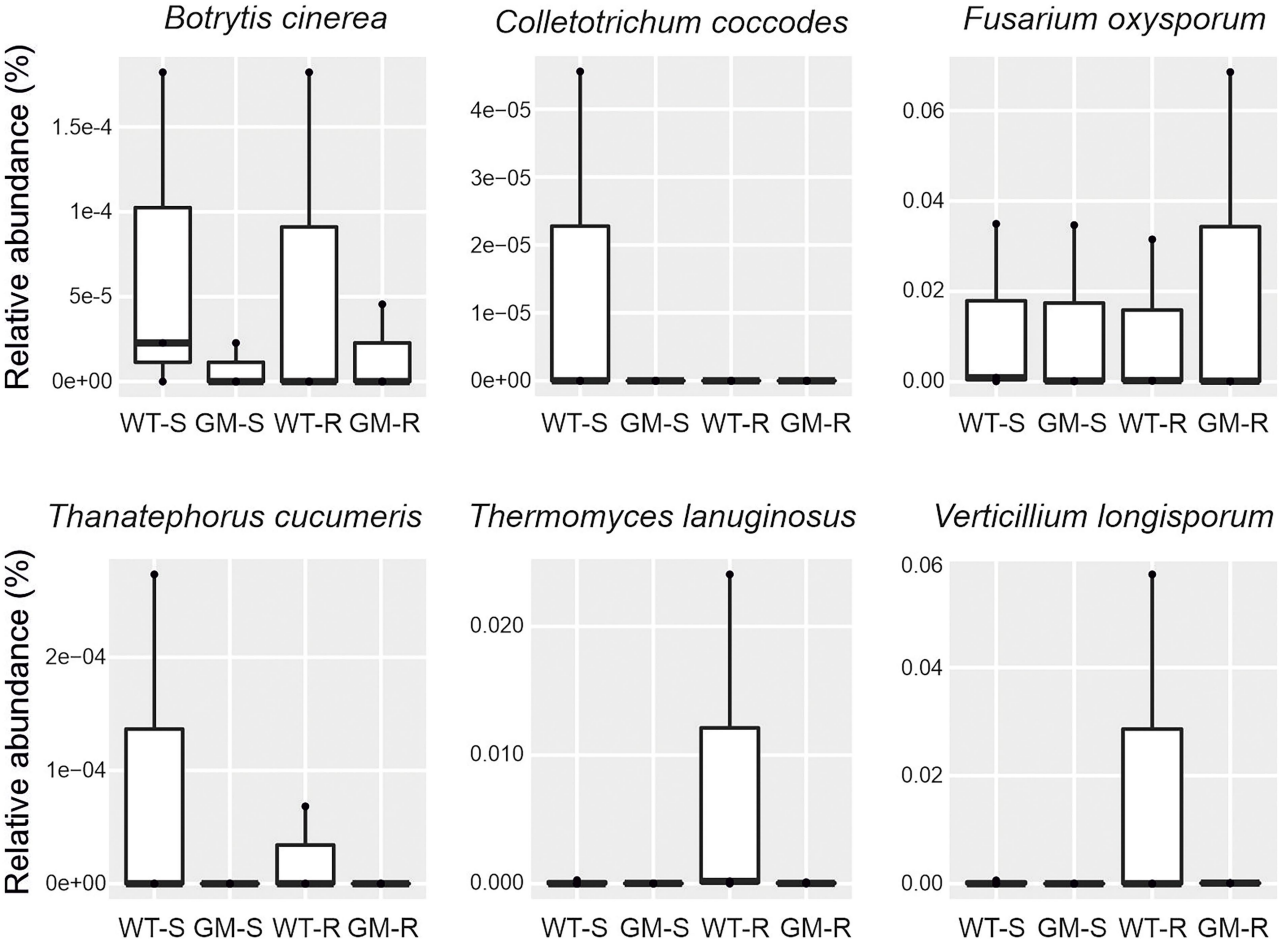
The relative abundance of fungal plant pathogen with assigned species level using FAPROTAX for four different group of samples. WT-S, Wild Type Surrounding soil; GM-S, Genetic Modified Surrounding soil; WT-R, Wild Type Rhizosphere soil; GM-R, Genetic Modified Rhizosphere soil.

The presence of plant pathogens in soil is an indicator of soil quality. The functional prediction by FAPROTAX revealed the same level of the possible plant bacterial pathogens in rhizosphere and surrounding soil of GM and wild type bamboo (Figures 7A,B). To evaluate the influence of GM bamboo cultivation on abundance of fungal pathogens, we annotated the plant pathogen related OTUs in different groups from fungal OTU taxonomy in species level with FUNGulid database. The relative abundance of six plant pathogens related OTUs (*Botrytis cinerea*, *Colletotrichum coccodes*, *Fusarium oxysporum*, *Thanatephorus cucumeris*, *Thermomyces lanuginosus*, *Verticillium longisporum*) were compared. Based on our analysis, although some plant pathogen related OTUs (*Colletotrichum coccodes*, *Thanatephorus cucumeris*, *Thermomyces lanuginosus*, *Verticillium longisporum*) were detected in soil microbiome of certain samples of wild type bamboo but did not present in those of GM bamboo (Figure 8). However, there was no significant increase on the pathogen abundances compared with soil microbiome of GM bamboo samples (*p* > 0.05; Figure 8). This result showed that overexpression of *Lc* gene in Ma bamboo did not lead to pathogen accumulation in soil.

## Discussion

Foreign gene transfer from GM bamboo may bring less concerns as its flowering period is normally much longer than most of other species. Yet, the potential underground changes caused by GM bamboo cultivation, especially the microbiota, are necessary to be carefully addressed for biosafety evaluation. The structure, diversity and function of soil microbiome are the key indicators in biosafety evaluation of transgenic plants. Whether GM plants will negatively impact the soil microbiome is in highly debate (Bruinsma et al., 2003; Guan et al., 2016). Some studies suggested that foreign gene products from GM plants negatively affect the abundance and diversity of soil microbial communities. Particularly, GM disease-resistant plants with antimicrobial products might inhibit the growth of beneficial microbe in soil (Rasche et al., 2006; Faragová et al., 2011). However, more studies revealed that the cultivation of GM plants had no effect or minor effect on soil microbial communities (Bruinsma et al., 2003; Guan et al., 2016). In this study, we showed that GM bamboo had no significant effect on the diversity and abundance of both bacteria and fungi in rhizosphere soil (Figure 3), suggesting that the soil microbial communities that closely attached to bamboo roots were not affected by GM bamboo. This finding agreed with many previous studies, which suggested that cultivation of GM plants did not change or negatively affect the soil microbial communities (Guan et al., 2016). Interestingly, studies demonstrated that other common factors, such as plant cultivar., soil type and development stage, have more power to influence the structure and functions of rhizosphere bacterial communities than genetic modification (Rasche et al., 2006).

Though the functions of microbiota are hardily to validate, multiple software are developed to predict biological and ecological functions (Louca et al., 2016; Nguyen et al., 2016; Douglas et al., 2020; Sansupa et al., 2021). We investigated the predicted functions for microbiota itself (basic metabolisms) and fitness effects on host plants (nutrition uptakes, presence of pathogens). Specifically, multiple processes of both fungal and bacterial communities kept at similar levels, involving in microbiome basic metabolisms, nitrogen usage and percentage of plant pathogens (Figure 6). For the basic metabolism profiling, it was probably that the root exudates of GM bamboo were mostly the same as those of wild type bamboo, since anthocyanin accumulation occurred only within bamboo cells (Xiang et al., 2021). Nitrogen fixing bacteria, as the key modulators of rhizosphere soil, might contribute greatly for plant production (Igiehon and Babalola, 2018). The nitrogen usage, including nitrogen fixation, was also comparable among groups, which indicated that the amount of available nitrogen fixed by microbes were equivalent from GM and wild type bamboo cultivation.

Plants can recruit beneficial microbes to rhizosphere for protection against pathogens (Mendes et al., 2011; Yu et al., 2022). The abundance estimation of plant pathogen is an indicator for such ability. Thus, we predicted the presence of plant fungal pathogens in species level with manual checking of FAPROTAX analysis, since the functional prediction in genera level are normally unreliable. The abundance of both bacterial and fungal plant pathogens had no significant difference (Figures 7, 8), indicating that GM bamboo cultivation would not enrich the abundance of investigated plant pathogens. In addition, four species of plant pathogens (*Colletotrichum coccodes*, *Thanatephorus cucumeris*, *Thermomyces lanuginosus*, *Verticillium longisporum*) were detected in the surrounding/rhizosphere soil of wild type bamboo but not in GM bamboo. A possible explanation could be that GM bamboo with overexpression of maize *Lc* gene and increased anthocyanin accumulation had an accelerated antioxidant activity itself (Xiang et al., 2021), which might result in an inhibited growth of existing pathogens. To confirm our elucidation, however, a test of artificial soil-borne pathogen inoculation for bamboo tolerance is required as a future study.

As to the surrounding soil, the bacterial diversity and abundance of GM bamboo increased significantly while fungal diversity and abundance decreased slightly with comparison to those of wild type bamboo (Figure 3). This shift might lead to minor changes of bacterial functions in aerobic chemoheterotrophy, aromatic compound degradation, nitrate reduction, and human associated. More experiments are needed to be performed to answer the questions on why GM bamboo affect the change of bacterial communities in surrounding soil, as well as the persistence of this effect. Moreover, based on the administration policy on transgenic plants in China, the GM bamboo could only be grown in the restricted greenhouse conditions currently, a long-term observation on soil microbiome in the field will provide more information in the future.

This is, to our knowledge, the first report of analyzing the effect of GM bamboo on soil microbiota. The influence of GM plants on rhizosphere microbial assembly and function are variable, depending on foreign gene product and many other factors (Faragová et al., 2011; Guan et al., 2016; Zhang et al., 2021; Li et al., 2022). The accumulation of anthocyanin in cells of *Lc* gene overexpressed bamboo had no dramatically change in the structure, abundance and functions of rhizosphere soil microbial communities, although have slight changes in surrounding soil. Regarding to the biosafety issue of GM plant, our results indicated that there was no negative impact of rhizosphere soil microbial communities for GM bamboo. What we could conclude was based on our current techniques and knowledge, and we believe that strict and continuous monitoring of any GM plant cultivation is needed practically, therefore, further long-term experiments to evaluate the situation in field with natural condition are necessary.

## Conclusion

In this study, we evaluated the impact of GM bamboo cultivation on the soil microbiota. The structure, abundance and function of both bacteria biota and fungal biota on rhizosphere and surrounding soil from GM and wild type Ma bamboo cultivation were analyzed. Our results indicated that there was no substantial alteration on rhizosphere soil microbial communities for GM and wild type Ma bamboo. Though minor change of microbial structure and abundance was observed for surrounding soil, the predicted functions including basic metabolism, nutrition usage and pathogenicity were kept at comparable levels. We proposed that growth of GM bamboo has no negative effect on rhizosphere and surrounding soil microbiota.

## Data availability statement

The data presented in the study are deposited in the NCBI SAR repository, accession numbers SRR20962121-SRR20962144.

## Author contributions

QZ conceived the study. KW, ML, CC, and SC performed the experiments. KW, ML, and QZ analyzed the data and wrote the manuscript. XM and CL co-supervised this project. All authors read and approved the submission of this manuscript.

## Funding

This work was supported by Natural Science Foundation of Fujian Province (2022J02023), Forestry Science and technology program of Fujian Province (2022FKJ06), National Natural Science Foundation of China (31870660 and 32071847), and the Foundation for the Development of Forestry Research in Fujian Agriculture and Forestry University (118/72202200201) to QZ, as well as Forestry Peak Discipline Construction Project of Fujian Agriculture and Forestry University (72202200205).

## Conflict of interest

The authors declare that the research was conducted in the absence of any commercial or financial relationships that could be construed as a potential conflict of interest.

## Publisher’s note

All claims expressed in this article are solely those of the authors and do not necessarily represent those of their affiliated organizations, or those of the publisher, the editors and the reviewers. Any product that may be evaluated in this article, or claim that may be made by its manufacturer, is not guaranteed or endorsed by the publisher.

## Footnotes

^1^https://www.grandviewresearch.com/industry-analysis/bamboos-market
