# Supplementary figures and images for: The impact of genetic modified Ma bamboo on soil microbiome
Source: Front Microbiol. 2022 Nov 1;13:1025786. doi: 10.3389/fmicb.2022.1025786 (PMC9664077; doi:10.3389/fmicb.2022.1025786)

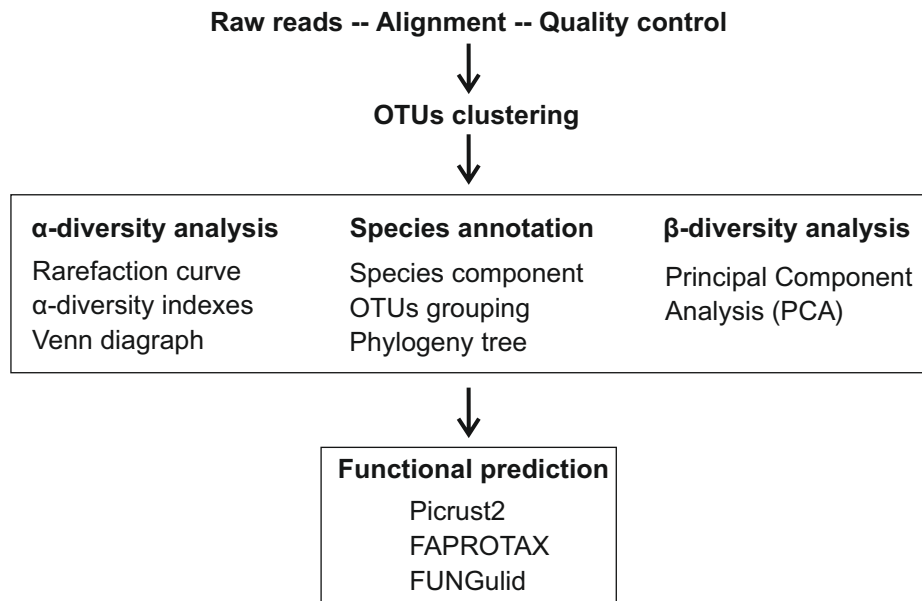

Supplementary Fig1. Workflow of the amplicon sequencing data analysis.

Supplement: Supplementary file 3 [file Image_1.pdf]
