# Supplementary material for: The impact of genetic modified Ma bamboo on soil microbiome
Source: Front Microbiol. 2022 Nov 1;13:1025786. doi: 10.3389/fmicb.2022.1025786 (PMC9664077; doi:10.3389/fmicb.2022.1025786)
Supplement: Supplementary file 4 [file Image_2.pdf]

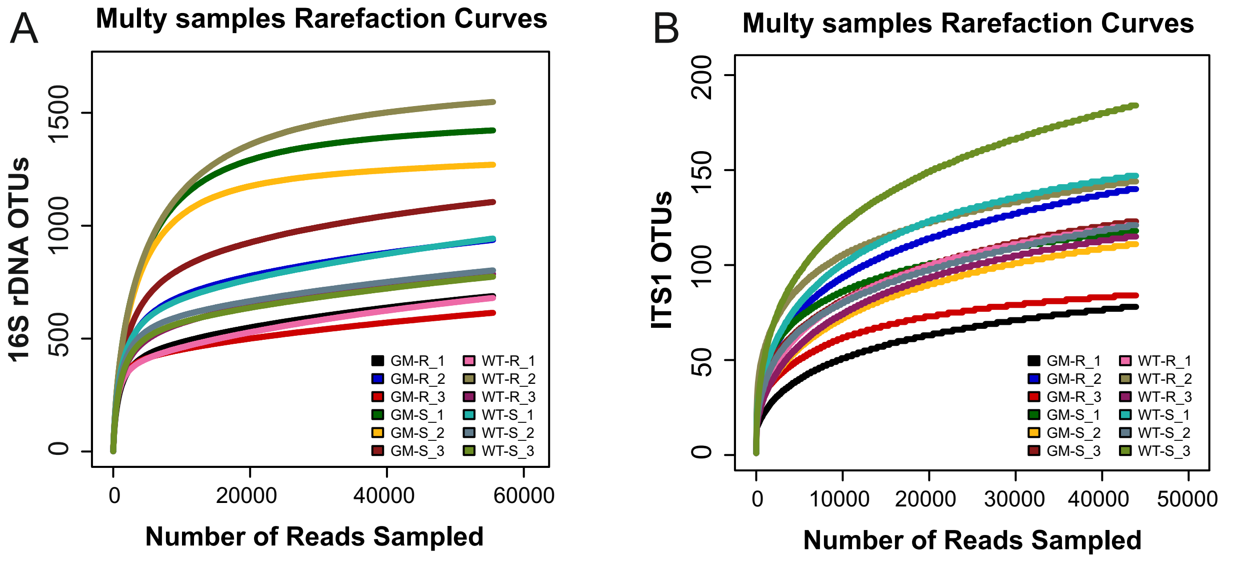

Supplementary Fig2. Rarefaction curves of OTUs from A) 16S rDNA and B) ITS1 vs the number of sequence reads sampled in this study.
